# Unusual Hydatid Cysts: Cardiac and Pelvic-Ilio femoral Hydatid Cyst Case Reports and Literature Review

**DOI:** 10.21470/1678-9741-2019-0153

**Published:** 2020

**Authors:** Nazim Kankilic, Mehmet Salih Aydin, Tansel Günendi, Mustafa Göz

**Affiliations:** 1 Department of Cardiovascular Surgery, Medical School of Harran University, Şanlıurfa, Turkey.; 2 Department of Pediatric Surgery, Medical School of Harran University, Şanlıurfa, Turkey.

**Keywords:** Hydatid Disease, Heart, Pelvic, Iliac, Cardiopulmonary Bypass

## Abstract

**Introduction:**

Hydatid cyst is a parasitic disease caused by *Echinococcus granulosus*, most commonly seen in the liver and lungs. The hydatid cyst is rarely seen in the heart and iliofemoral region, representing less than 2% of all cases. In this article, we report our cases of hydatid cysts in unusual loci.

**Methods:**

Between 2015 and 2018, 6 rare cases of hydatid cysts were diagnosed at the Cardiovascular Surgery Department of Harran University. Four of these patients had cardiac localization and two patients had their cysts located in the iliofemoral region, extending to the pelvic zone. All patients were female. Three patients had no other organ involvement. One patient with cardiac hydatid cyst underwent normothermic cardiopulmonary bypass + total pericystectomy + Cooley-like aneurysmectomy. Total pericystectomy was performed in three other patients with intrathoracic locus by normothermic cardiopulmonary bypass. Two patients who were referred to our clinic with palpable iliofemoral mass were evaluated with appropriate imaging methods and diagnosed accordingly. Multiple iliofemoral cysts were managed with pericystectomy and drainage by a single incision made over the inguinal ligament.

**Conclusion:**

Hydatid cyst disease can develop in cardiac chambers and inguinal region with or without hepatic or pneumatic involvement. Normothermic cardiopulmonary bypass can be safely used in patients with cardiac hydatid cysts, and capitonnage similar to ventricular aneurysm repair in patients with a widely involved cystic lesion can be very useful for the protection of ventricular functions.

**Table:** 

Abbreviations, acronyms & symbols	
AV	= Atrioventricular
CT	= Computed tomography
ELISA	= Enzyme-linked immunosorbent analysis
IHA	= Indirect hemagglutination
MRI	= Magnetic resonance imaging
PCR	= Polymerase chain reaction
US	= Ultrasound

## INTRODUCTION

Hydatid cystis parasitic infection mainly caused in humans by *Echinococcus granulosu*s^[1]^. It is a significant disease in some parts of the world, especially in South America, Southern Europe, Australia, New Zealand, Africa, Turkey, India and the Middle East region^[1,2]^. People are infected by taking *Taenia* eggs orally. The disease is seen as cysts in various organs. Hydatid cysts can occur with symptoms of pain or mass effect. It is most commonly seen in the liver (75%), then in the lungs (15%) and the rest of the body (10%)^[3,4]^. Other organs, such as spleen, heart and brain, are rarely affected^[5,6]^. Bone and soft tissue hydatidosis accounts for approximately 1-5% of all cases^[7,8]^.

### Epidemiology and Serology

In the *Echinococcus* transmission cycle, there is an intermediate host and a main host. People are accidentally exposed to the disease. The adult worm is usually found in the small intestine of the main host. Every day, thousands of eggs can be released. These discharged eggs are taken by an intermediate or accidental host. Oncospheres in eggs taken from the intestinal mucosa of the host enter the circulation. When they reach the liver or other organs by circulation, they form cysts that grow and produce protoscolices. While the main host is eating infected meat, protoscolices are reintroduced into the intestinal mucosa and converted into adult worms^[9,10]^. The life cycle is 4-7 weeks. For the formation of adult worms, a main host and an intermediate host are required. Human-to-human transmission is not proven to be possible^[10]^.

Serology is useful for diagnosis. Initial screenings are indirect hemagglutination (IHA) and enzyme-linked immunosorbent analysis (ELISA) for IgG, IgM or IgE antibodies. The IHA test is positive in more than 80% of liver hydatid cysts. Further serological tests include immunoblotting and immunoelectrophoresis. Polymerase chain reaction (PCR) is used especially in epidemiological studies^[11]^.The biopsy involves situations where it is impossible to diagnose by clinical or laboratory investigations alone. Open biopsy with simultaneous excision of cysts is the preferred method with a large resection site^[12,13]^. Preoperative percutaneous aspiration can be performed in most cases, although it is not necessary to directly visualize protosclerosis to confirm the diagnosis. Although not so common, anaphylaxis is among the complications of a cyst infection, or pneumonia and hemobilia during the procedure^[14]^.

## METHODS

In this article, we discuss the surgical treatment of unusual cases of cardiac and pelvic iliofemoral hydatid cysts (between 2015 and 2018) and the literature review. A detailed physical examination was performed in all patients. Their histories were taken, and host contact was questioned in detail. Detailed blood tests of patients were studied. Histopathological examinations were performed in all patients who were considered compatible with hydatid cyst. A large number of giant cells, necrotic tissue and the surrounding membrane structure were present in the basophilic laminar structure along with germination membranes. Electrocardiography, brain tomography, thoracic and abdominal computed tomography (CT), echocardiography, cardiac magnetic resonance imaging (MRI), lower extremity color Doppler ultrasound (US), pelvic MRI and lower extremity MRI were used for the diagnosis of hydatid cysts. The medical therapy was arranged before surgery. Albendazole treatment was given to all patients at least 4 days before surgery and treatment continued at least 3-6 months after surgery.

In our operations, a median sternotomy/appropriate incision, depending on the coexisting cysts, was performed under general anesthesia. Normothermic cardiopulmonary bypass was applied with or without beating-heart technique, dependent on the location of the cyst. After bicaval/unicaval and aortic/femoral cannulation, atriotomy/ventriculotomy was carried out, exposing the localized cyst. The cyst structure and germination membrane were removed without cyst content spillage. The atrial/ventricular cavity was irrigated with hypertonic saline solutions and closed with running Prolene sutures. Coexisting cysts in brain, liver and lung were managed with their respective surgical clinics. No recurrence was detected in any patient in the 4-week follow-up.

## CASE REPORTS

Demographic data of all patients, host contact, involvement in other regions and information about the surgery are summarized in Table1.

**Table1:** Demographic data, host contact, involvement and surgery information.

	Age	Gender	Host contact	Localization	Cyst size	Other involvement	Incisions and cannulations	Operation techniques
Case 1	21	Female	+	Left ventricle	41 × 32 mm	-	Median sternotomy + aortic and two-stage (unicaval) venous cannulation	Normothermia + CPB+ Cooley-like aneurysmectomy + cystectomy
Case 2	28	Female	+	Right atrium	25 × 28 mm	Lung	Median sternotomy + aortic and bicaval venous cannulation	CPB + beating-heart+ cystectomy
Case 3	26	Female	+	Left ventricle	11 × 13 mm	Lung and brain	Right thoracotomy+ right femoral artery and bicaval venous cannulation	Normothermia + CPB + cystectomy
Case 4	17	Female	-	Right ventricle	20 × 21 mm	Liver and lung	Clamshell incision + right femoral artery and bicaval venous cannulation	Normothermia + CPB+ cystectomy
Case 5	35	Female	-	Iliofemoral(right)	Multiple cysts83 × 67 mm	-	Over the inguinal ligament	Total pericystectomy
Case 6	12	Female	+	Iliofemoral(right)	Multiple cysts165 × 113 mm	-	Over the inguinal ligament	Total pericystectomy

CPB=cardiopulmonary bypass

### Case 1

**Fig. 1:**
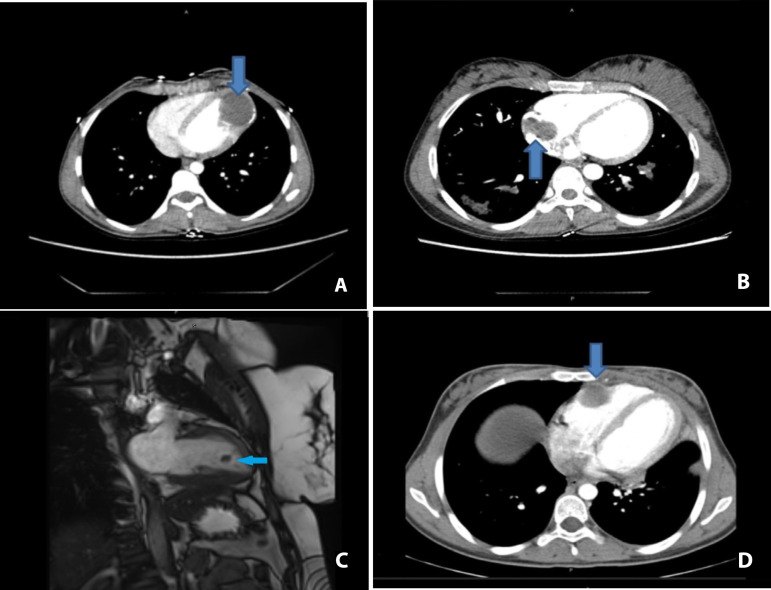
CT and MRI images of cardiac hydatid cyst.

**Fig. 2:**
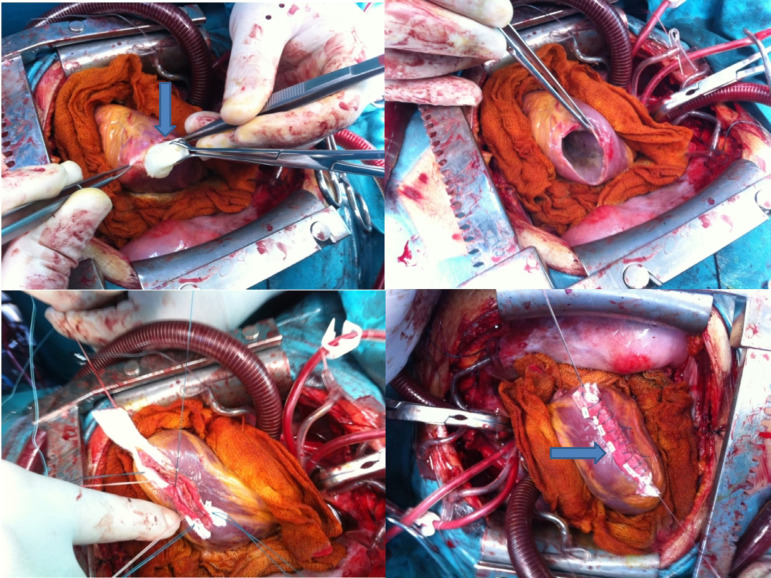
Cardiac hydatid cyst surgery-capitonnage similar to ventricular aneurysm repair.

A 21-year-old woman was admitted to hospital for palpitation, chest pain and shortness of breath. Transthoracic echocardiography showed a single hypoechoic cyst in the left ventricle. There was no communication between the cardiac chambers, but the cyst has shifted the left ventricle to the right. The size of the cyst was 45×50 mm. Chest CT scan showed a cystic hypodense lesion (41×32 mm) in the apicolateral region of the left ventricle (Figure 1A). There were no hydatid cysts in the liver, lungs and other organs on radiological examination. Under general anesthesia, a median sternotomy was performed. Aortic and two-stage (unicaval) venous cannulations were performed. Cardiopulmonary bypass was applied with normothermia. On exploration, a 41×32 mm cyst was detected in the anterolateral aspect of the left ventricle. The area of the cyst was isolated from the rest of the heart and from the pericardial space. The hydatid cystic material was aspirated and the cystectomy was performed. The cavity formed by removal of the cyst mass was irrigated with 20% hypertonic saline solution. There was no connection with the left ventricular cavity. After partial resection, the cyst cavity was closed between Teflon felt strips attached with two layers of horizontal mattress sutures using 2-0 Ethibond® (Ethicon, Johnson & Johnson Medical N.V., Belgium), in a Cooley-like aneurysmectomy (Figure 2). The patient was discharged on the 8^th^ postoperative day, with no subsequent problems.

### Case 2

A 28-year-old woman was admitted to hospital for shortness of breath. On cardiac examination, there was a diastolic murmur in mitral focus. Transthoracic echocardiography showed a 2.6× 2.9 cm septated hydatid cyst in the right atrium. Left ventricular function was normal. A 25 × 28 mm lesion associated with right atrial wall was detected on chest CT examination (Figure 1B). Under general anesthesia, a median sternotomy was performed. Aortic and bicaval venous cannulations were performed and cardiopulmonary bypass was applied with beating-heart technique. After cannulation, right atriotomy was carried out, exposing the localized cyst. The cyst structure was removed without cyst contents pillage. The right atrial cavity was irrigated with hypertonic saline solutions. The right atrial cavity was closed with a 4-0 Prolene suture. Supraventricular tachycardia developed on the 1^st^ postoperative day. After beta blocker therapy, sinus rhythm was restored. The patient was discharged on the 6^th^ postoperative day.

### Case 3

A 26-year-old woman was admitted to hospital for headache and shortness of breath. Transthoracic echocardiography revealed left ventricular lesion. Cardiac MRI revealed an 11× 13 mm lesion in the left ventricle (Figure 1C). This lesion was interpreted in favor of myxoma. The patient was prepared for operation for the current mass. A right thoracotomy was applied under general anesthesia. Right femoral artery and bicaval venous cannulations were performed. Cardiopulmonary bypass was performed with normothermia and right atriotomy incision was done. A hydatid cyst in the left ventricular apex was visualized with a camera from the mitral valve using a transseptal approach. The cyst was removed without damaging the surrounding tissues. Incised septum and right atriotomy were closed with 4-0 Prolene sutures. The patient was discharged on 6^th^ postoperative day with no further problems.

### Case 4

A 17-year-old woman was admitted to hospital with the complaints of shortness of breath, abdominal pain and abdominal swelling. Transthoracic echocardiography showed an 18 × 20 mm hydatid cyst in front of the right ventricle. On chest CT, there was a 20 × 21 mm hydatid cyst at the anterior of the right ventricle. Also, two hydatid cysts were at the upper lobe (43 × 28 mm) and lower lobe (20 × 13 mm) of the left lung, respectively (Figure 1D). Under general anesthesia, a left thoracotomy was applied and the aforementioned cysts in the left lung were removed by the thoracic surgery team, then we performed a clamshell incision to reach the mediastinum. Upon exploration, hydatid cyst was found on the anterior surface of the right ventricle, extending to the right ventricular cavity. Right femoral artery and bicaval vessels were cannulated. Cardiopulmonary bypass was applied with normothermia. The right ventricular wall was incised to remove the cyst. The cavity was irrigated with hypertonic solutions accordingly. Right ventricular wall was repaired with 4-0 pledgeted sutures. There were no complications in the postoperative period. The patient was discharged on the 5^th^ postoperative day.

### Case 5

**Fig. 3:**
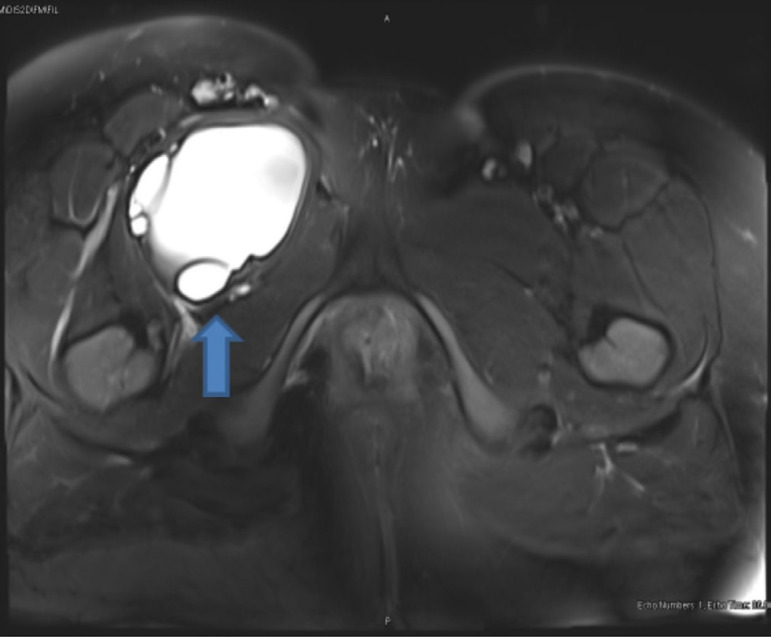
MRI image of pelvic iliofemoral hydatid cyst.

**Fig. 4:**
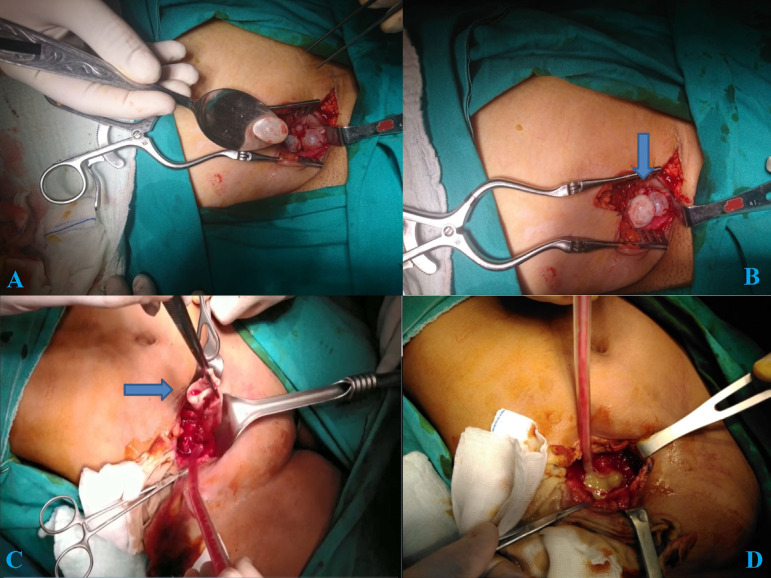
Pelvic iliofemoral hydatid cyst surgery.

A 35-year-old woman was admitted to cardiovascular surgery clinic for pain and swelling in the right leg. Physical examination and laboratory analyses were normal. The lower extremity color Doppler ultrasound showed insufficiency at the right saphenofemoral junction level. Pelvic MRI showed a hypodense, multicystic lesion (83 × 67 mm) in the right inguinal canal (Figure 3). Radiological examination demonstrated no hydatid cysts in the liver, lungs and other organs. Under general anesthesia, the iliac region was explored with incisions made over the inguinal ligament. After incision, a large number of hydatid cysts were found suppressing the iliac and femoral vascular structures of the iliac region. Total pericystectomy and drainage were performed (Figure 4A and B). Afterwards, iliac and femoral cavities were irrigated with appropriate hypertonic solutions and a hemovac drain was placed in the cyst cavity. Cavities were closed with single 4-0 Prolene sutures. The patient was discharged on the 5^th^ postoperative day.

### Case 6

A 12-year-old child was admitted to pediatric surgery clinic for right thigh pain and swelling. Lower extremity color Doppler ultrasound showed insufficiency at the right iliac and femoral vein level. A contrast-enhanced abdominal tomography revealed a 93 × 163 mm cystic lesion in the anteromedial segment of the right thigh, extending to the pelvic region retroperitoneally. The MRI of the right thigh and femur revealed a cyst lesion of approximately 165 × 113 mm in the widest area, extending from the pelvic region to the proximal anteromedial segment of the right thigh. There was no other organ involvement on radiological examinations. The patient was consulted a tour clinic subsequently. A cooperative surgery was planned. The iliac region was excised with the incisions made over the inguinal ligament under general anesthesia. Upon exploration, two hydatid cysts were observed in the iliac region, connected with each other, one extending to the femoral region. Total pericystectomy and drainage were applied to all cysts (Figure 4C and D). Iliac and femoral cavities were irrigated with hypertonic solutions and a hemovac drain was kept in the cyst cavity for 5 days. Follow-up was uneventful and the patient was discharged without subsequent problems.

## DISCUSSION

### Cardiac Hydatid Cyst

Hydatid cyst can reach the myocardium by the left side of the heart with coronary circulation. Cardiac hydatid cyst is most frequently seen in the left ventricle (55-60%). Other regions are interventricular septum (10-20%), right ventricular myocardium (15%), left atrium (8%), pulmonary artery (7%), pericardium (8%) and right atrium (3-4%)^[1]^. Cardiac involvement occurs initially by invasion of myocardium from the coronary arteries. The second involvement is due to pulmonary veins as a result of rupture of pulmonary echinococcal cysts^[15]^. The hydatid cyst is more common in the left ventricle due to the dominance of the left coronary artery^[16-18]^. Right ventricular hydatid cysts are different from left ventricular cysts. They tend to expand inside the right ventricular cavity and in the subendocardial space and are more prone to rupture, which can lead to pulmonary embolism, anaphylaxis and sudden death. Left ventricular hydatid cysts grow as subepicardial masses and rarely rupture in the pericardial space^[19,20]^.The endocardium is easily invaded by hydatid cyst for the obvious reason that endocardium does not give inflammatory reaction like the pericardium^[21]^. Septal cyst can cause atrioventricular (AV) block by conduction disturbance^[22]^. Intracardiac hydatid cyst adjacent to a valve may also lead to valve destruction, requiring valve replacement^[23]^. Generally, the symptoms of the hydatid cyst only occur when the cyst expands. Patient symptoms can include allergic reactions^[24]^, heart failure^[25]^, exertional angina^[26]^, arrhythmias^[27]^, heart block^[20,28]^, cardiac tamponade^[21]^ and sudden death^[29]^.

Echocardiography is noninvasive, highly sensitive, and easily performable diagnostic method in cardiac hydatid cysts. The hydatid cyst may occasionally be mistaken for cardiac tumors. In some cases, the differentiation between a myxoma and a hydatid cyst is made pathologically. CT and MRI are effective in localizing specific masses in the heart and making a differential diagnosis between tumors and hydatid cysts^[30]^. In radiological imaging, specific findings of hydatid cysts are calcification of the cyst wall, presence of daughter cysts, and membrane detachment. CT is reported to be the best imaging modality that shows wall calcification, whereas anatomic neighborhood of hydatid cysts, the status of the cystic structures and the exact anatomical position are best indicated by MRI^[29,31]^.

The main treatment for ventricular hydatid cyst is surgery^[32]^. The surgical technique includes median sternotomy and right or left side thoracotomy with the cardiopulmonary bypass, moderate hypothermia and aortic cross-clamping. Pulmonary artery should be clamped to prevent pulmonary embolism if the hydatid cyst is located on the right side of the heart. Cystopericystectomy is the gold standard procedure for the cyst, but a conservative approach (partial pericystectomy) is also used to protect organ functions^[32]^.Ventricular myocardial *echinococcosis* unrelated to the cardiac chambers can be operated without using cardiopulmonary bypass, as defined by Birincioglu et al.^[33]^.

We prefer the use of normothermic cardiopulmonary bypass in cases of hydatid cysts. In this way, we have shown that the normothermic cardiopulmonary bypass approach can be successfully used in hydatid cyst cases. In addition, in patients with hydatid cysts involving large ventricular area, capitonnage repair similar to ventricular aneurysm repair protects ventricular anatomy and functions. This repair technique has a positive effect on remodeling.

### Pelvic/Iliac/Femoral Hydatid Cyst

Rare anatomic locations reported for hydatid cysts are numerous. These include: breast, pancreas, adrenal gland, spleen, ovary, pleura, chest wall, cavernous sinus, thyroid, parotid gland, submandibular gland, peritoneal cavity, retroperitoneum, inguinal canal, muscle, bone, thigh, femoral region and subcutaneous tissue^[3,4,34-36]^. One of them is iliac and hydatid cysts affecting the femoral region. The hydatid cysts that develop in this localization cause thrombosis and insufficiency by decelerating the blood flow, compressing the vascular structures in the region^[37-39]^. Liver and lungs are also affected in patients with atypical cyst hydatid disease and explain the pathophysiology. However, pathophysiology is still not fully understood in patients with a typical localization without involvement in these organs. The pathophysiology of pelvic, femoral and iliac hydatid cysts without parenchymal organ involvement is still not clearly established. There are theories that hydatid embryos are located in these areas by hematogenous or lymphatic pathways^[40]^. The liver is the first filter to meet the hepatic portal flow. Many of the larvae are kept here. The larvae passing through the microvascular wall in the liver reach the lungs. In some patients, hydatid larvae can pass through the liver and lungs through the capillary system and it is assumed that any tissues and organs can be reached^[41,42]^. It is also thought that it can migrate into various intraabdominal organs and voids through transmural migration through the intestinal wall by passing through the venous mesenteric lymph vessels^[42]^.

US is the first diagnostic tool for detecting hydatid cyst disease in soft tissue. The detection of hydatid cysts in MRI and the determination of their neighbors is the best examination for surgical planning^[43]^. In such cases, imaging methods should be used wisely. This facilitates the identification of the incision site and the incision area. Instead of multiple incisions, it is possible to reach all cysts with a single and appropriate incision. This provides the patient with comfort, rapid postoperative recovery and shorter hospital stay. In our iliac/femoral cases, a single incision made from the top of the inguinal ligament ushered us to the exact location of the lesion.

Treatment options in these patients are medical, watch and wait approach and surgery. Surgery is the basis of hydatid cyst treatment^[44]^. Surgical treatment modalities were reported as radical surgery (total pericystectomy or partial resection of the affected organ), conservative surgery (open cystectomy) or simple tube drainage for infected hydatid cysts^[45]^. In cases of pelvic-iliac hydatid cysts involving large areas, the cystic mass tends to protrude to neighboring tissues due to high cyst pressure. This can be advantageously used in favor of patients with pelvic-iliofemoral hydatid cysts. However, these cysts are adjacent to important vascular and nerve structures, so the operation should be performed meticulously.

Before surgery, the patient should be treated with albendazole or mebendazole 400 mg twice daily for at least 4 days. Albendazole treatment should be continued for at least 1 month, and mebendazole treatment should be continued for at least 3 months^[46]^. The use of albendazole before surgery reduces the intracystic pressure and facilitates the operation, while treatment with albendazole decreases the risk of recurrent hydatid disease. Percutaneous treatment may be an alternative to single and uncomplicated cysts smaller than 5 cm. This procedure is contraindicated in divided complex cysts, cysts associated with the biliary system and cysts that are difficult to access^[14]^.

## CONCLUSION

Many studies have shown that hydatid cyst can affect many organs and tissues. A detailed investigation should be done in patients with hydatid cysts. It should also be kept in mind that hydatid cyst disease can affect the inguinal region and cardiac chambers, with or without hepatic or pulmonary involvement. Our cases are a small group of rare entity in this regard. In the surgical aspect, capitonnage similar to ventricular aneurysm repair is important for ventricular anatomy and remodeling. If it is failed to achieve, severe ventricular dysfunction may develop. Normothermic cardiopulmonary bypass should be considered as an effective method as moderate hypothermic bypass.

**Table:** 

Author's roles & responsibilities	
NK	Substantial contributions to the conception or design of the work; or the acquisition, analysis, or interpretation of data for the work; final approval of the version to be published
MSA	Substantial contributions to the conception or design of the work; or the acquisition, analysis, or interpretation of data for the work; final approval of the version to be published
TG	Substantial contributions to the conception or design of the work; or the acquisition, analysis, or interpretation of data for the work; final approval of the version to be published
MG	Substantial contributions to the conception or design of the work; or the acquisition, analysis, or interpretation of data for the work; final approval of the version to be published
